# Environmental and Genetic Factors Affecting Apospory Expressivity in Diploid *Paspalum rufum*

**DOI:** 10.3390/plants10102100

**Published:** 2021-10-04

**Authors:** Mariano Soliman, Marika Bocchini, Juliana Stein, Juan Pablo A. Ortiz, Emidio Albertini, Luciana Delgado

**Affiliations:** 1 Instituto de Investigaciones en Ciencias Agrarias de Rosario (IICAR), CONICET, Facultad de Ciencias Agrarias, Universidad Nacional de Rosario, Rosario S2125ZAA, Zavalla, Argentina; marianosoliman@gmail.com (M.S.); jstein@unr.edu.ar (J.S.); ortiz@iicar-conicet.gob.ar (J.P.A.O.); 2 Department Agricultural, Food and Environmental Sciences, University of Perugia, 06121 Perugia, Italy; marika.bocc87@gmail.com (M.B.); emidio.albertini@unipg.it (E.A.)

**Keywords:** apospory expressivity, inheritance, environment, diploid level, *Paspalum rufum*

## Abstract

In angiosperms, gametophytic apomixis (clonal reproduction through seeds) is strongly associated with polyploidy and hybridization. The trait is facultative and its expressivity is highly variable between genotypes. Here, we used an F_1_ progeny derived from diploid apomictic (aposporic) genotypes of *Paspalum rufum* and two F_2_ families, derived from F_1_ hybrids with different apospory expressivity (%AES), to analyze the influence of the environment and the transgenerational transmission of the trait. In addition, AFLP markers were developed in the F_1_ population to identify genomic regions associated with the %AES. Cytoembryological analyses showed that the %AES was significantly influenced by different environments, but remained stable across the years. F_1_ and F_2_ progenies showed a wide range of %AES variation, but most hybrids were not significantly different from the parental genotypes. Maternal and paternal genetic linkage maps were built covering the ten expected linkage groups (LG). A single-marker analysis detected at least one region of 5.7 cM on LG3 that was significantly associated with apospory expressivity. Our results underline the importance of environmental influence in modulating apospory expressivity and identified a genomic region associated with apospory expressivity at the diploid level.

## 1. Introduction

A small proportion of seed plants are able to avoid meiosis and fertilization, to produce seeds identical to their maternal parent, by apomixis [1,2]. This natural reproductive system owns great agronomic importance as it allows hybrid combinations to be perpetuated without the need to repeat the original crosses every season. It also accelerates breeding programs and enables seed propagation of crops reproduced vegetatively [1,2]. In this sense, apomictic plants are potentially a constant source of renewable seeds, which would be of great advantage for crop production, especially for developing countries, if their uses became freely available to the public and private sectors [3,4].

Gametophytic apomixis in nature is strongly associated with polyploidy and almost all gametophytic apomictic are polyploid while their sexual relatives are typically diploid [5,6]. Gametophytic apomixis involves the formation of unreduced embryo sacs from the megaspore mother cell itself after avoiding meiosis (diplospory) or from a nucellar cell of the ovule (apospory) [7,8]. In both cases, the embryo originates by parthenogenesis of the non-reduced egg cell. In addition, the endosperm formation may require the fertilization of polar nuclei (pseudogamy) or develop autonomously [9]. Although apomixis is widely distributed among angiosperms, it is rare in crop gene pools. Despite its presence in wild relatives of some cereal crops, introgression approaches had failed to transfer the character [10,11]. For this reason, the transformation of sexual crops into apomictic ones is a long-awaited dream of plant breeders to harness the potential of apomixis [1].

Natural gametophytic apomictic species have been extensively studied. Some previous work has shown that this trait is inherited as a single dominant locus which has been confirmed in several aposporic apomictic systems such as *Paspalum* [12,13], *Pennisetum*, *Cenchrus* [14], *Panicum* [15], *Ranunculus* [16], and *Hypericum* [17,18]. Numerous studies have detected a great number of markers closely linked to the trait, revealing that the apospory-specific genomic region (ASGR) includes a large chromosomal segment with strong repression of recombination [19,20,21,22]. In addition, some meiotic abnormalities were detected in apomictic genotypes, which were assigned to the presence of genetic rearrangements [19,20,21,22,23]. These aspects, which have also been extensively reported for several apomictic species, such as *Pennisetum* [24,25], *Cenchrus* [26,27], *Hieracium* [28,29], *Panicum* [30], and *Brachiaria* [31], have made positional cloning of the key genes responsible for apomixis difficult. Otherwise, comparative expression analysis between apomictic and sexual genotypes of natural apomictic species identified many genes associated with different apomixis components [32,33,34,35,36,37,38,39,40,41,42,43,44].

Despite the knowledge acquired, the transference of the full trait to sexual species is still pending. However, current breeding programs in apomictic species provide empirical evidence of the use of apomixis to fix superior hybrid genotypes and also reveal the problems related to its application. *Paspalum notatum*, is a native grass to South America with important forage uses that has been bred by hybridization, taking advantage of its apomixis capacity to fix genetic combinations [45,46]. These works reported that crosses between sexual and apomictic genotypes generate a low proportion of apomictic progeny compared to sexual progeny and point out the difficulties of producing highly apomictic genotypes with superior agronomic traits, due to the great variability of apomixis expression [45,46]. These observations are consistent with the different levels of apomixis expression reported in *Boechera* [47]. Moreover, some authors have proposed that the quantitative occurrence of apomixis in *Hieracium* is modulated by additional unlinked genetic factors [29,48,49], and in *Eragrostis curvula*, QTL analysis of diplospory expressivity revealed the presence of two main QTLs separated from the diplospory locus [50].

The fact that the same genotype can reproduce either by apomixis or by sexuality is possible because most apomictic polyploids are facultative and both processes coexist in the same plant or even in the same ovary [16,51]. Previous work in *P. malacophyllum* proposed that in facultative apomictic tetraploids, both reproductive pathways are unstable at the beginning of female development, but finally, only apomixis succeed [52]. In contrast, certain species of the genus *Paspalum* are able to produce AES at the diploid level, but seeds are only produced by sexuality [53,54,55,56,57,58]. In this context, it was proposed that apomixis emerges from a rearrangement of sexual developmental programmes, by asynchrony in gene expression timing, induced by polyploidization and or hybridization [59,60,61,62,63]. In addition, it was also reported that the proportion of sexuality and apomixis could be influenced by environmental conditions [64,65]. Thus, according to previous results, genetic/epigenetic background, ploidy level, as well as environmental effects would influence the switch between apomixis and sexuality. The identification of quantitative genetic components, controlling the expressivity of apomixis, would be a great molecular tool that would allow the early identification of superior highly apomictic hybrid genotypes in breeding programs [66].

*Paspalum rufum* Nees, is organized in agamic complex composed of sexual diploids (2*n* = 2*x* = 20) and facultative apomictic tetraploids (2*n* = 4*x* = 40), [58,62]. A comprehensive characterization of natural diploid populations, from the North-East region of Argentina, identified some genotypes capable of producing viable AES that form seeds either by sexuality (B_III_ hybrids) or by apomixis [67,68]. These findings corroborated the presence of genetic determinant(s) of apomixis at the diploid level, as had been previously suggested for this species [56]. Subsequently, the reproductive characterization of experimental diploid populations of *P. rufum*, generated by crossing genotypes with low proportions of AES, revealed that apospory was transmitted to almost all progeny. Moreover, it was possible to increase the proportion of ovules producing AES by both hybridization and polyploidization of diploid genotypes [69].

To better understand the variability of apospory expressivity the present work proposes a diploid model of *P. rufum* to study its transgenerational transmission and its behavior under environmental variations. Furthermore, we develop a genetic linkage map that would facilitate the mapping of qualitative and quantitative loci that influence apomixis expressivity at the diploid level.

## 2. Results

### 2.1. Reproductive Characterization of the F_1_ Population

The F_1_ progeny used in this work was previously generated by crossing two natural diploid genotypes, (R6#45 x R5#49) [69], both capable of producing AES to similar degrees, 5.77% and 13.3%, respectively [68], (Table 1). The phenotypes (%AES) that are part of this F1 population (39 individuals), hereafter named F_1-C_, and of both parental genotypes, were previously determined in Corrientes, North-East of Argentina [68,69].

**Figure 1:**
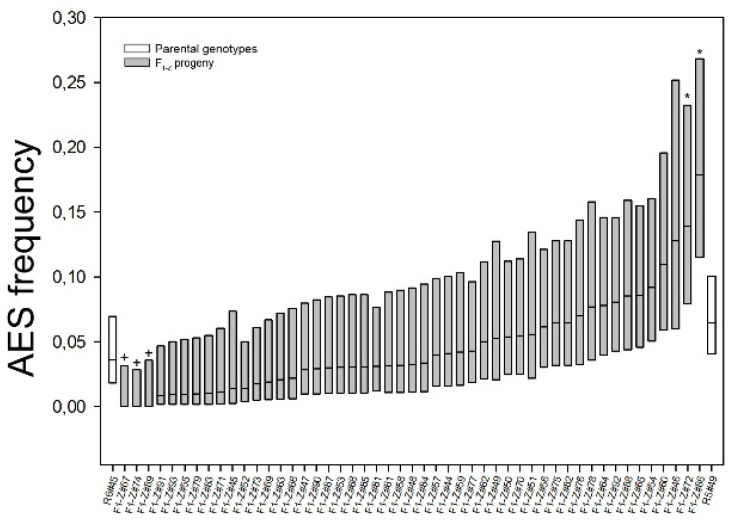
Apospory expressivity of the parental genotypes R6#45 and R5#49 (white boxes), and the F_1-Z_ population (grey boxes), determined in Zavalla. The frequency of ovaries containing AES (box middle line) scored in each plant is plotted together with 95% confidence intervals (CI). The asterisks mark the hybrids that showed significant difference (*p* < 0.05) with respect to paternal genotype (*p* < 0.05), + symbol indicates genotypes without AES.

In the present work, 49 additional hybrids of the population (hereafter named F_1-Z_), were characterized, together with the parental genotypes, in Zavalla, in the central region of Argentina (Figure 1 and Table 2). Reproductive classification (%AES) was performed by cytoembryological observation of cleared ovaries at anthesis. Parental genotypes, R6#45 and R5#49, characterized in Zavalla, showed 3.6% and 6.4% of AES, respectively, while in the F_1-Z_ apospory expressivity ranged from 0% to 17.8% (mean: 4.56% ± 3.82) (Figure 1 and Table 2). The comparative analysis between each hybrid of the F_1-Z_ and the parental genotypes revealed that the %AES of most of the hybrids was included within the range of both parental genotypes values (Figure 1). However, higher apospory expressivity was detected in two plants (#72 and #86) which showed a significant increase (13.75% and 17.71%, respectively) with respect to both parental genotypes. On the other hand, three individuals: #67, #74, and #89 did not show any AES (Figure 1 and Table 2). Considering all F_1_ plants ((F_1-C_ and F_1-z_), 84 hybrids (95.45%), out of 88 showed AES and only four hybrids (4.54%) had exclusively MES.

**Figure 2:**
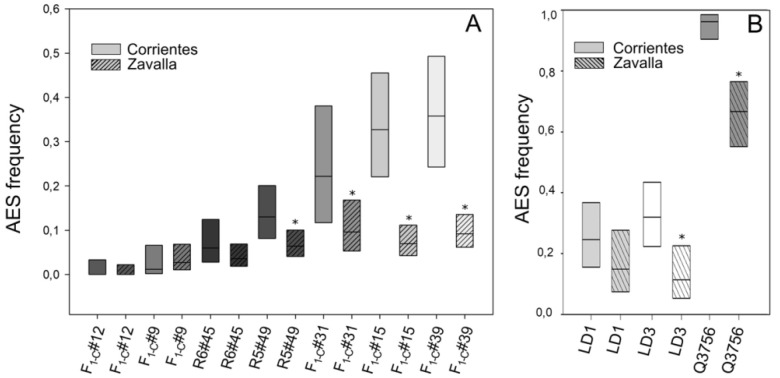
Apospory expressivity in *P. rufum* (**A**) diploid genotypes and (**B**) tetraploid individuals, assessed at the two locations. The frequency of ovaries containing AES (box middle line) scored in each plant is plotted together with 95% confidence intervals (CI). Boxes colored with different shades of grey indicate different genotypes; plain boxes represent measurements made in Corrientes, while striped boxes represent measurements made in Zavalla. Asterisks mark significant differences of the same genotype between different locations.

In agreement with previous results [69], the F_1-Z_ showed a wide range of variation of apospory expressivity, indicating possible transgressive segregation of the trait as a result of hybridization. The comparison of the average apospory expressivity between both F_1_ populations showed that the F_1-Z_ presented a significant reduction compared to F_1-C_ (Table 2). In addition, the parental genotypes also showed a reduction in %AES when moved from Corrientes to Zavalla (Figure 2, Table 1 and Table 2). Specifically, the maternal genotype (R6#45) changed from 5.8% to 3.6% and the paternal genotype (R5#49) decreased significantly from 13% to 6.4% (Figure 2, Table 1 and Table 2).

### 2.2. Evaluation of the Environmental Influence on Apospory Expressivity

Following the observations described above, we analyze the stability of apospory expressivity in the two environments assayed. Therefore, five genotypes with different %AES were selected from the F_1-C_ population to be evaluated at both Zavalla and Corrientes. Two diploid hybrids with low and no apospory expressivity (F_1-C_#9 and F_1-C_#12, respectively) and three with relatively high expressivity (F_1-C_#31, F_1-C_#15, and F_1-C_#39) were selected as well. Moreover, tetraploid genotypes with different apospory expressivity were also included: one highly apomictic natural tetraploid Q3756 and two artificially generated tetraploids LD1 and LD3 (Table 1).

The apospory expressivity of the different individuals in Corrientes and Zavalla is shown in Figure 2. Statistical analysis revealed that apospory expressivity decreased significantly in hybrids #31, #15, and #39. The %AES decreased from 22.2%, 32.7% and 35.8% in Corrientes (Table 1) to 9.6% (Supplementary Materials Table S1), 6.9% and 9.2 in Zavalla (Table 3), respectively (Figure 2A). Meanwhile, individuals with low apospory expressivity maintained similar low proportions in the two locations, #12 retained 0% and #9 showed 1.22% and 2.76%, in Corrientes (Table 1) and Zavalla (Table 4), respectively. The tetraploid genotypes showed also a reduction in %AES, when grown in Zavalla. LD1 decreased from 31.9% (Table 1) to 11.3% (Supplementary Materials Table S1), LD3 from 24.6% (Table 1) to 14.9% (Supplementary Materials Table S1), and Q3756 from 96.1 (Table 1) to 66.7 (Figure 2B and Table 1 and Supplementary Materials Table S1).

**Figure 3:**
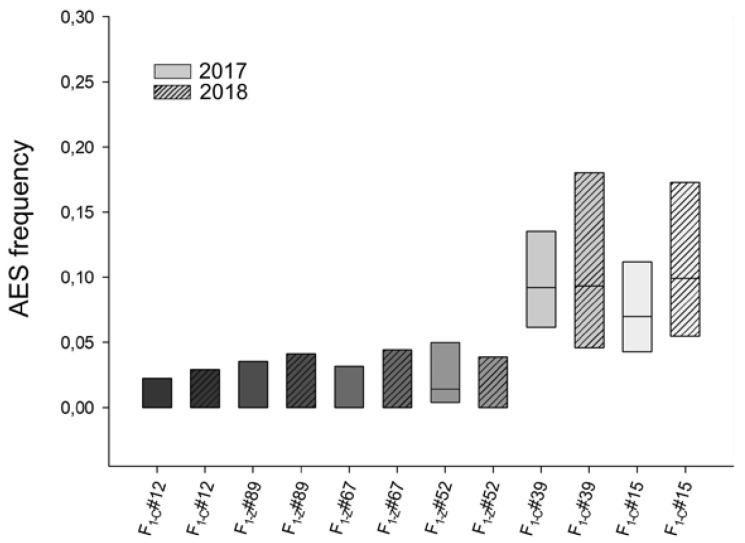
Apospory expressivity in diploid individuals of *P. rufum* measured during two annual periods in Zavalla. The frequency of ovaries containing AES (box middle line) scored in each plant is plotted together with 95% confidence intervals (CI). Boxes colored with different shades of grey indicate different genotypes; plain boxes represent measurements made during 2017, while striped boxes represent measurements made during 2018. No significant differences were detected between the two years.

On the other hand, to restrict the environmental variation, some genotypes were evaluated in the same location (Zavalla) but over different years. Four diploid hybrids with low %AES (F_1-C_#12, F_1-Z_#89, F_1-Z_#52, and F_1-Z_#67) and two with a relatively high proportion (F_1-C_#15 and F_1-C_#39) were used (Figure 3). No significant variations were detected between years; genotypes F_1-C_#12 (Table 4), F_1-Z_#89, and F_1-Z_#67 showed fully sexual ovaries in both years while F_1-Z_#52 only showed AES in the first evaluation (1.41%) but it only showed MES in the second year (Figure 3, Table 1 and Supplementary Materials Table S1). Genotypes with a higher proportion of AES showed similar values in both determinations, F_1-C_#15 presented 6.97% and 9.9%, while F_1-C_#39 showed 9.2% and 9.33% in both periods (Figure 3 and Table 3).

### 2.3. Apospory Expressivity Variation in F_2_ Progenies

To analyze the effect of hybridization on apospory expressivity, two F_2_ progenies obtained by crossing two pairs of plants from the F_1-C_ population with similar (low or high) apospory expressivity were generated. Thus, F_1-C_#9 × F_1-C_#12 (mean 1.40% ± 1.98, Table 4) and F_1-C_ #15 × F_1-C_#39 (mean 8.79% ± 1.81, Table 3) were crossed to obtain the families F_2L_ and F_2H,_ respectively. The apospory proportion of both populations was determined for two consecutive years at Zavalla. The characterization revealed no significant difference between both periods for each individual, only one hybrid (F_2H_#8) showed a significant variation in the %AES between both years (Table 3 and Table 4). 

**Figure 4:**
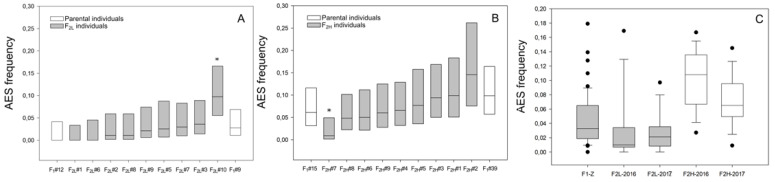
Frequency of apospory expressivity of the two F_2_ offspring measured during 2017 (**A**) F_2L_ and (**B**) F_2H_. The parental genotypes are in white boxes and hybrids in grey boxes. Asterisks indicate a significant difference (*p* < 0.05) of the progeny compared to the parental genotypes (*p* < 0.05). The frequency of ovaries containing AES (box middle line) scored in each plant is plotted together with 95% confidence intervals (CI). (**C**) Apospory expressivity variation of F_1-Z_ (2016), F_2L_, and F_2H_ progenies (2016 and 2017). Boxes represent the 95% confidence intervals (CI), the middle lines indicate the median value, outliers are marked by black circles, and different colors indicate significant differences between median values (*p* < 0.05).

Individuals of each F_2_ progeny were compared to their parental genotypes. Samples fixed at Zavalla in the same year (2017) were used for this analysis, except for F_1-C_#9 which was only analyzed in 2018. In F_2L_, the mean %AES of hybrids along both years showed a range of variation from 0% to 13.3% AES (Table 4), and most individuals were not significantly different from the parental genotypes (Figure 4A). However, the plant F_2L_ #10 showed a significant increase in apospory expressivity with respect to its progenitors (Figure 4A, Table 3). In F_2H_, all individuals produced AES, the mean %AES, over both years, ranged from 1.82% to 15.61% (Table 3). The comparison of the %AES, between the parental genotypes, and each hybrid during 2017, revealed that most hybrids were not different from the parental genotypes (Figure 4B), only F_2H_ #7 showed a significant reduction in apospory expressivity (0.9%) with respect to parental genotypes (Figure 4B and Table 3). Overall, these results verify the stability of the trait across different years and showed that, in the two F_2_ progenies, as well as in the F_1_, the parental phenotypes seem to delimit the range of %AES variation of the progeny. Notably, the F_2H_ family, which descend from both aposporous parents with higher proportions of AES, showed, in both years, mean values of %AES (mean: 10.05 and 7.19, median: 10.8 and 6.5 respectively) (Table 3, Figure 4C), significantly higher than the means of the F_2L_ population (mean: 3.33 and 2.75, median: 1.0 and 2.1) (Table 3, Figure 4C), which derive from parents with only one aposporous parental genotype, with low %AES (Figure 4C). Moreover, the F_2H_ median value was also significantly higher than the mean proportion of apospory expressivity of the F_1-Z,_ (mean: 4.60, median: 3.26), which come from both parents (R6#45 and R5#49) with intermediate %AES (Table 1, Figure 4C).

### 2.4. F_1_ Genetic Linkage Map

To develop molecular tools to study apospory inheritance and identify genomic regions associated with apospory, we decided to build a genetic linkage map diploid *P. rufum*. At first, to select informative AFLP markers, 105 selective primer combinations were tested on parental genotypes. This analysis identified 33 highly polymorphic combinations, each producing more than 15 polymorphic fragments (Supplementary Materials Table S2) that were selected for linkage analysis in the F_1_ progeny (Supplementary Materials Figure S2). Good-quality AFLP libraries were obtained for 87 out of 88 F_1_ offspring (F_1-Z_#90 was discarded). The hybrid origin of F_1_ plants was confirmed for all 87 individuals included in the analysis. A binary data matrix was built for each parental genotype containing 278 maternal, 239 paternal, and 110 bi-parental (segregating from both parental genotypes) markers (Table 5). Markers fitting the expected segregations, 1:1 for SDAF (single dose amplification fragment) and 3:1 for BSDF (bi-parental single dose fragment), were used for mapping. Segregation analysis confirmed the expected values (0 < χ^2^ < 3.8) for 266 maternal, 221 paternal, and 94 BSDF markers (Table 5). About 7.4% of the markers showed a variable degree of distorted segregation ratios in the progeny (4.1 < χ^2^ < 104.3) (Table 5). Two linkage maps corresponding to the maternal and paternal parents were constructed.

**Figure 5:**
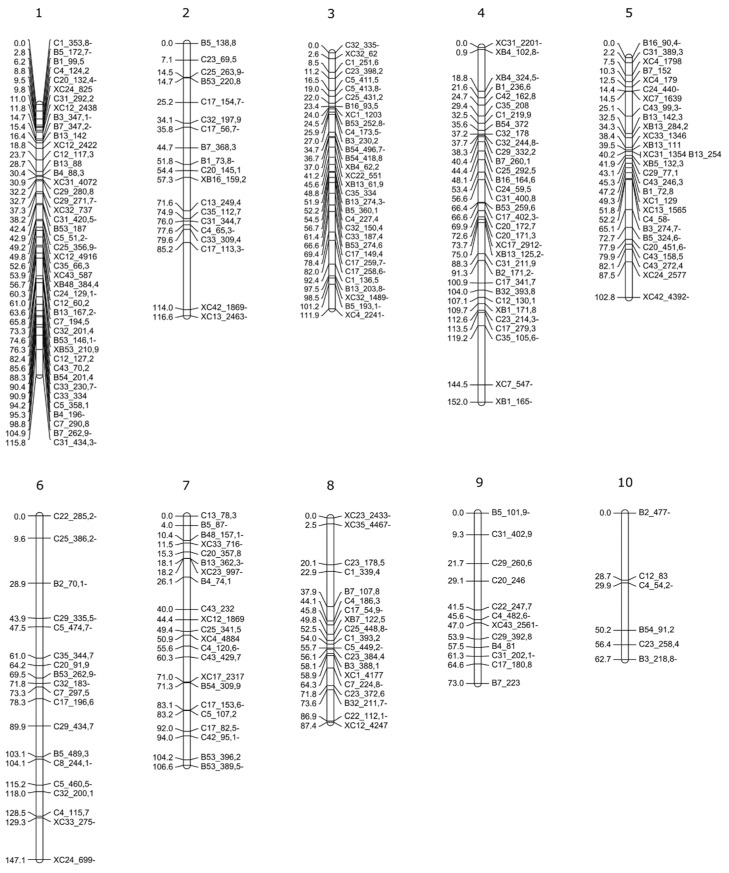
Linkage map of the diploid (2*n* = 2*x*) maternal genotype of *P. rufum* (R6#45). Markers and distances in cM (Kosambi) are indicated on the right and left, respectively. Each co-segregation group includes SDAF and BSDF (starting with an X) markers in coupling and repulsion-phase (-).

The data matrix of the maternal progenitor (R6#45), including a total of 360 markers (266 SDAF and 94 BSDF), was first filtered and thus 75 markers were discarded due to similarity and two individuals were removed due to 96.5% of genetic similarity. As described in the Section 4, an initial grouping was assayed at LOD > 6.0 which fixed the order of 219 markers, with a mean distance of 7.5 cM (±6.4), in 20 LG in the coupling phase. A second test at LOD > 2.0, using the above-fixed order, added 35 additional markers, extending the total distance from 1478.4 cM to 1672.0 cM and shortening the average inter-markers distance to 7.14 cM (±6.5). In the next step, maternal homologous LGs were combined, including markers that segregated in the repulsion phase, as described in the Materials and Methods. Finally, the maternal map incorporated 234 markers distributed in 10 LGs, covering a total distance of 1071.8 cM with a mean inter-marker distance of 4.78 cM (±4.8 cM) and a maximum distance of 28.74 cM. Each LG contained an average of 23.4 (±11.7) markers with a maximum of 44 and a minimum of six markers. The largest LG, LG4, covered 151.9 cM and contained 33 markers. The shortest was LG10, which covers 62.7 cM and included 10 markers (Figure 5 and Supplementary Materials Table S3).

**Figure 6:**
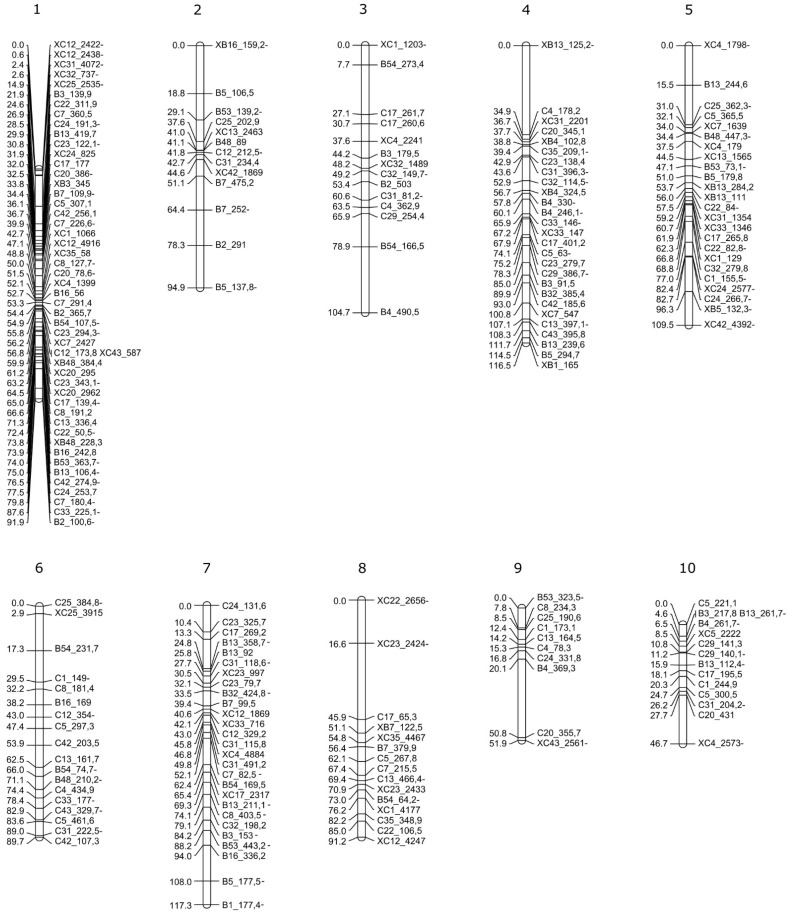
Linkage map of the diploid (2*n* = 2*x*) paternal genotype of *P. rufum* (R5#49). Markers and distances in cM (Kosambi) are indicated on the right and the left respectively. Each co-segregation group includes SDAF and BSDF (starting with an X) markers in coupling and repulsion-phase (-).

In the paternal (R5#49) map, the initial matrix included a total of 315 markers (221 SDAF and 94 BSDF) (Table 5) and after filtering 74 markers were discarded due to their similarities. Following the same procedure as for the maternal map, the first approach ordered 185 markers in 20 LGs, covering 965.8 cM and a mean inter-marker distance of 5.85 cM (±5.3). The next step at LOD > 2 added 29 additional markers, extending the total distance to 1193 cM and the mean distance between markers to 6.15 cM (±6.3). Finally, after combining homologous LGs, the paternal map included 212 markers, distributed in 10 LGs, covering a total distance of 914 cM. The average distance between markers was 4.53 cM (±5.4) reaching a maximum distance of 34.9 cM. Each LG contained an average of 21.2 (±11.8) markers, LG1 was the largest with 50 markers, covering 117.2 cM, and LG10 which was the shortest containing 14 markers distributed over 46.7 cM (Figure 6, Supplementary Materials Table S3).

**Figure 7:**
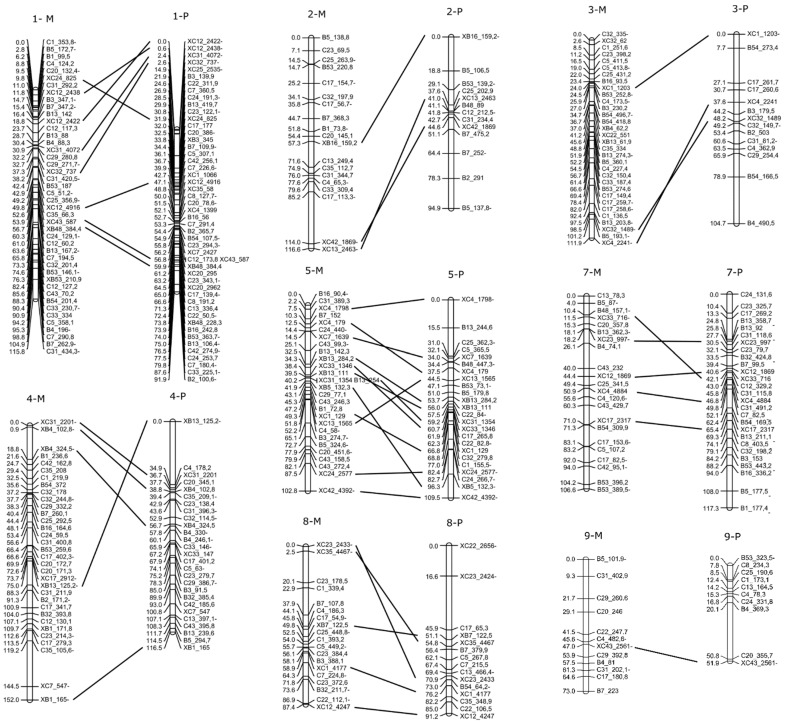
Maternal (M) and paternal (P) homologous chromosomes identified by BSDF (starting with an X). Marker’s names and distances in cM (Kosambi) are indicated on the right and the left, respectively. The relative order of BSDF markers between both maps is indicated by horizontal lines.

Based on bi-parental markers segregation, it was possible to identify eight out of the ten female and male homologous LGs (Figure 7, Supplementary Materials Table S4). As there were no bi-parental markers on the LG6 and LG10 numbers, they were arbitrarily assigned (Figure 5 and Figure 6). The relative order between bi-parental markers in both maps showed that most of them are equally ordered in maternal and paternal maps (Figure 7).

### 2.5. Identification of Genomic Regions Associated with Apospory Expressivity

A linear regression analysis was performed combining phenotypic information of F_1_ hybrids, as the dependent variable, and genotypic data derived from the mapping procedure, as the independent variable [70,71]. Due to the variation of the %AES between locations, F_1-C_ and F_1-Z_ populations were analyzed separately. This single-marker analysis detected a total of 40 significant associated markers (*p* < 0.05) with an R^2^ from 0.1 to 0.34. The evaluation of the F_1-C_ showed 26 markers associated with apospory expressivity (0.1 < R^2^ < 0.34), 23 of which were included in the map. As three of them had been previously eliminated by similarity, their position specifications correspond to those similar mapped markers (B16_164.6, C29_332.2, C32_244.8), the other three remaining unlinked (Table 6). As shown in Table 6, the LGs with the highest number of associated markers were: LG3, with a total of seven markers, six maternal (M), and one paternal (P), and LG4 with a total of 10 markers, seven M, one P and two bi-parental (BP). The other LGs containing associated markers were: LG2 (one marker), LG5 (one marker), and LG10 (three markers). The markers with the highest R^2^ (>0.15) were found in LG2, LG3, and LG4 (Table 6).

Regarding the F_1-Z_ population, 13 markers showed a significant association (*p*<0.05) with R^2^ ranging from 0.10 to 0.17, 12 markers were mapped, one of which was assigned by similarity as mentioned above (C12_83), (Table 7). As for the F_1-C_, LG3 showed the highest number of associated markers, with a total of 5 markers (two M, two P, and one BP), while LG6 showed two paternal markers, LG7 two maternal markers, and LG1, LG8, and LG10 showed one marker each (P, M, and M, respectively) (Table 7). The markers with the highest R^2^ (>0.15) were found in LG3 and LG7.

**Figure 8:**
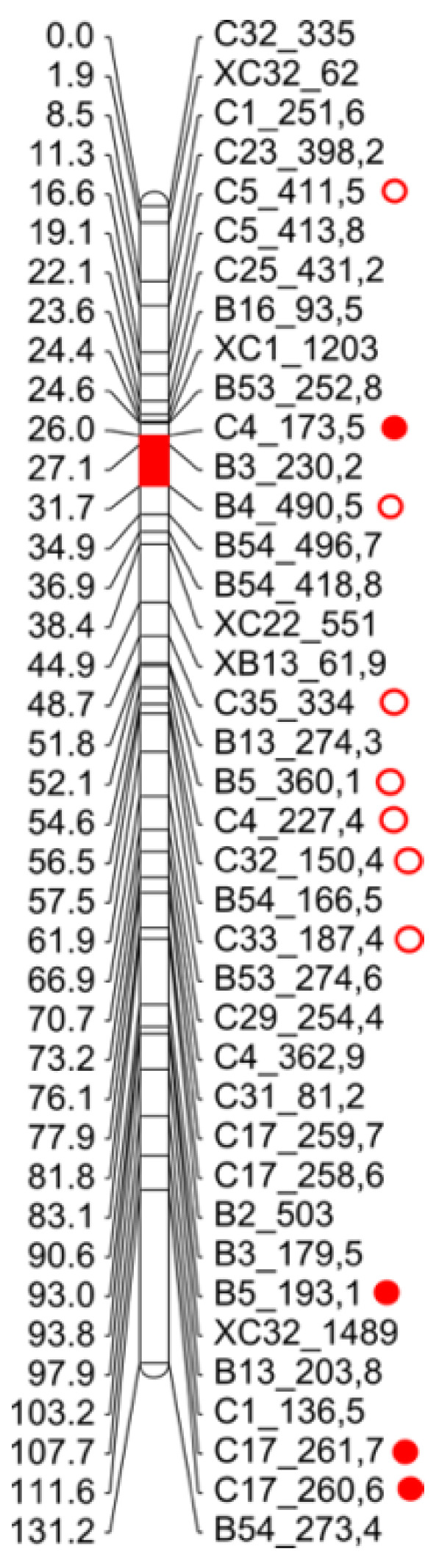
Detail of the LG3 obtained after combining maternal and paternal LG3s. The circles indicate the markers associated with %AES, the open circles indicate the markers found in the F_1-C_, and the filled red circles are those found in the F_1-Z_. The red color on the LG marks a region delimited by a marker detected in F_1-C_ and others detected in F_1-Z_.

Interestingly, both F_1_ populations showed markers associated with the trait on LG3 and also on LG10, but the latter has an arbitrary number in both parental maps as it was not possible to identify female and male homologous. Then, focusing on LG3, both maternal LG3 and paternal LG3 were combined using the “*Combine Groups for Map Integration*” function (JoinMap 4.0) to characterize the relative order between the two groups of markers. The integration generated a new LG3 of 131.2 cM with a total of 39 markers. Most of the markers associated with apospory expressivity, identified from each population, clustered in different regions of the LG3, however, one marker from the F_1-C_, B4_490.5 (R^2^ = 0.13; *p* = 0.03) and another from the F_1-Z_, C4_173.5 (R^2^ = 0.12; *p* = 0.02) enclosed a region of 5.7 cM (Figure 8).

## 3. Discussion

The inheritance and genetic analysis of gametophytic apomixis have long been carried out mainly from a qualitative perspective, on natural polyploid systems, leading to the inherent difficulties of the ploidy level and also limiting the understanding of the variation of apomixis expressivity. *Paspalum rufum* offers a diploid system to study the inheritance of apomixis that would avoid the problems related to polyploidy. In the current work, we studied apomixis from a quantitative perspective by analyzing the variation of apospory expressivity in two consecutive generations of *P. rufum* diploid hybrids, and in different environments. In addition, a genetic linkage map was built and used for searching genetic regions that could be modulating the extent of the trait.

### 3.1. Apospory Inheritance

Although the main purpose of our work was to analyze the expressivity of apospory, it is necessary to discuss how apospory capacity is transmitted to the offspring at the diploid level. Our results showed a stable inheritance of the capacity to produce AES over two generations. In the F_1_ population, 84 individuals out of 88 were able to produce AES, and also in both F_2_ progeny, almost all hybrids were able to produce AES. As in F_1_, both parents are natural aposporic genotypes, and their genetic origin is unknown, we could not rule out the hypothesis of a single dominant gene, in homozygous or multiallelic configuration, which would confer aposporic ability to all progeny. In this sense, individuals considered as sexual would also carry aposporic potential but in a low proportion that is difficult to detect. This could be explained by the fact that inter-annual analyses revealed that some genotypes classified as completely sexual one year were able to produce a very low proportion of ovules with AES another year. In this context, our observation would be consistent with previous genetic analyses on the inheritance of apomixis at the tetraploid level in the genus *Paspalum*, which mainly point to a single dominant locus involved [7,12,13]. Then, the genetic determinant(s) of apospory would be constitutively present in the F_1_ offspring of *P. rufum*, but the expressivity of the trait would be modulated by other unknown internal and/or external factors.

### 3.2. Apospory Expressivity Variation

Based on the results described above, two main questions arise: how the environment influences apospory expressivity and whether genetic/epigenetic background determines the degree of apospory expressivity. To start unravelling these issues concerning the first question, we analyzed the behavior of the trait in different environmental conditions. Our analyses have shown that when the same genotypes were grown in different locations, they showed significant variation in the degree of apospory expressivity. Several studies have reported variation in apospory under different circumstances. It was previously found that in facultative apomictic tetraploid genotypes of *P. notatum*, the frequency of apomixis was found to vary during the flowering period, being higher at peak flowering time and decreasing until the end of the flowering season [65]. Similar variations had been reported for highly apomictic wild *P. notatum* and *P. cromyorrhizon*, where apomictic reproduction varies throughout the flowering period [64,72]. Furthermore, tetraploid genotypes of *P. cromyorrhizon* were exposed to different day length treatments and the increment of light exposure from 12 to 14 h increased SES and decreased AES production [64]. Accordingly, a similar analysis in facultative apomictic *Ranunculus auricomus* reported that an extended photoperiod resulted in a higher proportion of sexual ovules [73]. Therefore, the authors concluded that environmental conditions, which affect flowering, may also influence the expression of apospory [64] and that residual sexuality could occur under less favorable environmental conditions [65]. A recent study on the sexual versus apomixis expression in *P. intermedium* reported that sexual reproduction increased at lower temperatures in facultative apomictic plants [74]. Regarding our results, we must point out that the main changes between Corrientes and Zavalla locations are the latitude and average temperature. These differences are associated with extended photoperiods [75] and lower temperatures [76], throughout the flowering months (from October to December) in Zavalla with respect to Corrientes. In addition, during sample fixation, along the years of our study, we noticed that there was a delay in flowering time at Zavalla with respect to Corrientes (data not shown) from the end of September to late October/early November, which also implies longer days. Thus, our observations reinforce previous results showing that apospory expressivity, in diploid and tetraploid genotypes of *P. rufum*, is significantly affected by environmental variations. As *P. rufum* plants are native to Corrientes [58], reallocation to Zavalla could result in a stressful condition that induces sexuality, which is consistent with the previously reported effect of stress on reproductive development in other apomictic systems [77,78]. Despite the location (latitude) effects, our work also showed that apospory expressivity in *P. rufum* is stable across the years when plants remain at the same location.

To deepen our understanding of the genetic/epigenetic influence on apospory expressivity, we analyzed the quantitative variation of the trait across successive generations. To rule out environmental influences on the trait, all plants were maintained in the same location. Then, two crosses were performed one between F_1_ hybrids with low apospory and the other between hybrids with high apospory, generating two F_2_ progenies, F_2L_ and F_2H_, respectively. We observed that the differences detected between the parents were maintained between the two F_2_ progenies so that the expressivity of the parental apospory was transmitted to the progeny and was also stable over different years. Accordingly, apospory expressivity in the F_1-Z_ population also showed low ranges of variation, similar to those of F_2L_, in line with the expressivity of its parents. A similar study was carried out in *Ranunculus* where apospory expressivity was analyzed in both F_1_ and F_2_ progenies. These results revealed that apospory expressivity was significantly increased in F_2_ progeny when both F_1_ parents, rather than just one, were aposporous [79]. In another study in *Hieracium*, on the assessment of the autonomous endosperm formation, it was revealed that the trait is conferred by a single dominant locus but additional genetic factors modulated its expressivity [44]. These outcomes are also in agreement with our previous results, where we found that both autopolyploidy and hybridisation of diploid *P. rufum* genotypes, induced higher expressivity of the trait [69]. Consequently, we propose that the degree of apospory expressivity, in the diploid system of *P. rufum*, is transmitted to successive generations in a dose-dependent manner and could be modulated by genetic/epigenetic factors. Regarding the latter, a recent comparative study of epigenetic patterns between the F_1-C_ hybrids used as parental plants for F_2L_ versus those used for F_2H_ revealed that the differential apospory expressivity of these genotypes was associated with different methylation patterns [80]. Thus, our observations of the transgenerational transmission of apospory expressivity could be associated with the fact that in plants, epiallelic variation can be stably propagated over several generations [81,82].

From an evolutionary perspective, our results are in agreement with a recent analysis of *P. intermedium* populations suggesting that environmental modulation of sexuality and apomixis expression would provide genetic variability to facultative apomictic linages allowing them to adapt to ecological challenges [74]. Furthermore, the effect we observed of hybridization on apospory expressivity would favor polyploidization within natural diploid sexual populations through the triploid bridge. This would indirectly help to establish an apomictic cytotype, as recently proposed by Hojsgaard et al. [63].

### 3.3. Linkage Map

To better understand the genetic basis modulating the expressivity of apospory at the diploid level, we built a genetic linkage map with AFLP markers. Two genetic linkage maps were generated for each parental genotype. The size of maternal and paternal maps were 1071.8 cM and 914 cM, respectively, which were similar to the maps of related diploid species such as *Paspalum notatum*, (991 cM) [83], *Setaria italic* (964 cM) [84], and *Sorghum bicolor* (1095 cM), [85]. Both linkage maps had ten LG which corresponds to the basic chromosome number of *P. rufum* (*n* = *x* = 10). The bi-parental markers identified eight of the ten homologous LGs between the two maps and also confirmed the relative order of the markers.

The maternal and paternal maps presented a mean distance between markers of 4.8 cM and 5.8 cM, respectively. These values are comparable and even lower, than those obtained in the previous map of *Paspalum notatum* [22,83] indicating a good relative density. The DNA content of *P. rufum* is 0.75 pg/Cx [57] which is equivalent to 733.5 Mbp, according to the 1pg/978 Mbp ratio previously reported [86]. Consequently, considering the total length (cM) of both recombination maps, 1 cM would be equivalent to a mean of 743.4 kpb. These are expected values for plant genomes and comparable to those estimated for the diploid *P. notatum* map (0.57 pg/Cx) in which 1cM is equivalent to 559 kpb [83]. Additionally, we found that the recombination frequency was similar for the maternal and paternal genotypes in contrast to the higher recombination frequency found in the genetic linkage map of paternal apomictic tetraploid *P. notatum* [22].

The map generated will be useful in future works to map markers linked to apomixis that have been previously found in other *Paspalum* species. It can also be useful to search for markers associated with parthenogenesis, as one of the parental genotypes of the F_1_ is capable of completing apomixis [68].

### 3.4. Quantitative Approach

Apospory expressivity regulation is in itself a very important aspect to take into account as in *P. notatum* breeding programs, sexual × apomictic offspring show a high range of variation in apospory expressivity [66,87]. Several works have dealt with the problem of obtaining a low proportion of highly apomictic hybrids after crosses between sexual and apomictic progenitors [45,46,66,87]. Therefore, although molecular markers linked to apospory are very useful in saving time and resources in breeding programmes, the identification of obligate apomictic hybrids with high agronomic performance is still limited [66]. These led us to analyze apospory expressivity from a quantitative perspective. Some research works have already considered apospory as a quantitatively modulated trait [29,48,49,50]. For *P. rufum* we observed that the F_1_ generation shows a wide range of apospory variation [69], in addition, some progeny genotypes showed a significant increase in apospory expressivity compared to the parental genotypes, which is in agreement with transgressive segregation [88]. This behavior could be attributed to recombination between parental genotypes, which have quantitative trait loci (QTL) with antagonistic effects [89]. A similar variation of apospory expressivity was previously reported in hybrids of *Paspalum notatum* [46] and *Hieracium* [29], leading the authors to suggest that unknown factors segregate in the genetic background modifying apospory expressivity. In line with these observations, recent comparative gene expression work between apomictic and sexual genotypes of *Ranunculus auricomus* reported that the pattern of gene expression was reflective of transgressive and genome dosage effects, supporting the hypothesis of a dominant factor controlling apomixis and modulated by secondary modifiers [90].

In this context, we looked for genetic factors that would be modifying apospory expressivity in the mapping population of *P. rufum*. We performed a single-marker analysis between markers and apospory expressivity. As we saw that different locations affect apospory expressivity, F_1_ sub-populations were analyzed separately. This initial analysis showed that the markers significantly associated with apospory expressivity were scattered across several LG. In F_1-C_, associated markers were able to explain 10–34% of the phenotypic variation, and LG3 and 4 had both the highest number of markers and the highest R^2^. In F_1-Z_, the number of significantly associated markers was about half of those found in F_1-C_, with a lower range of R^2^ from 10–17%. The wide dispersion of markers along the genome was in line with recent work in *Eragrostis curvula* where diplospory has, for the first time, been assessed as a quantitative trait. Although qualitative analysis confirmed that diplospory is determined by a single dominant locus, QTL analysis revealed that diplospory expressivity could be regulated by five different regions, two very closely linked to diplospory locus and three additional regions distributed along two different LG [50].

We observed that each of the F_1_ sub-populations showed different sets of associated markers, which could be explained by the strong influence of the environment on apospory expressivity. However, some of them coincide in the LG3, which had a total of 14 markers in both F_1-C_ (8) and F_1-Z_ (6). The integration of maternal and paternal LG3s showed that the markers found in each location clustered in different genomic regions. Notwithstanding, a marker detected in Zavalla was close (5.7 cM) to another marker detected in Corrientes, so this region could be considered in future analyses as a putative constitutive QTL.

The low proportion of phenotypic variation explained by the associated markers could be attributed to the low range of variation in apospory expressivity. This is evidenced by the difference observed between the two sub-populations as F_1-C_, which ranges from 0 to 35%, had both a higher number of associated markers and higher R^2^ values than F_1-Z_ which ranges from 0–17%. On the other hand, the small size of each sub-population could also have a negative impact on the results. Both aspects should be taken into account in future analyses. The molecular tools developed here will be the starting point for future molecular analyses of apomixis in the diploid system of *P. rufum*.

## 4. Materials and Methods

### 4.1. Plant Material

The starting plant material was the F_1_ progeny previously developed from the cross between two natural diploid genotypes: R6#45 used as pistillate parent and R5#49 used as pollen donor [69] (Table 1). The 39 individuals of the F_1_ offspring (named F_1-C_ in advance) had been previously established in the IBONE experimental field (Latitude: −27.469213. Longitude: −58.830635) and their reproductive mode had already been determined by cytoembryological observation [69]. In the present work, 49 additional individuals were added to the original F_1-C_ population, increasing its size to 88 individuals, which were established at the Agronomy Collage of the National University of Rosario, located in Zavalla (FCA-UNR, Latitude: −33.018538. Longitude: −60.878858) (named F_1-Z_ in advance). F_2_ progenies were developed by crossing F_1-C_ individuals, selected by their apospory expressivity. Two individuals with low apospory expressivity: F_1-C_#9 (1.2% AES) and F_1-C_#12 (0% AES) and two with high apospory expressivity: F_1-C_#15 (32.8% AES) and F_1-C_#39 (35.8% AES) were crossed as previously described [69], to generate F_2L_ and F_2H_ progenies respectively. Both F_1-C_#9 and F_1-C_#15 were used as pistillate parents, while the other two individuals as pollen donors, respectively (Table 1). Filled seeds were germinated in sterilized soil, and seedlings were planted in small pots in a greenhouse. Subsequently, the plants were transferred to a field nursery and grown under natural conditions at the FCA-UNR. To verify the hybrid origin of F_1_ and F_2_ plants and avoid individuals generated by self-fertilization, apomixis, or un-intended cross-contamination marker segregation analysis was performed. F_1_ and F_2_ progenies were evaluated by AFLP (see below) and RAPD markers respectively. A total of 13 primers of the UBC series were tested and five (UBC301, UBC310, UBC329, UBC344, and UBC349) showed polymorphism between the F_1-C_ genotypes used as parental genotypes of the F_2_ progenies (Supplementary Materials Figure S1, Tables S5 and S6). Three of the five polymorphic markers detected were used to assess the hybridity of F_2L_ and four for F_2H_. Each plant was scored for the presence of paternal and maternal bands and was considered hybrid if it showed at least two pollen donors specific bands (Supplementary Materials Figure S1 and Table S6). The data confirmed the existence of non-maternal offspring and parental combinations in the F_2_ generations (Supplementary Materials Table S6). In addition, previously characterized tetraploid genotypes were used, one natural (Q3756) and two artificially obtained by colchicine treatment, LD1 and LD3 [69], (Table 1).

### 4.2. Cytoembriological Analysis

The %AES of each genotype was determined by counting the number of ovaries containing AES, in anthesis inflorescences. Samples were treated according to Soliman et al., (2019) [91]. Briefly, they were initially fixed in FAA (70% ethanol: glacial acetic acid: formaldehyde in the proportion 90:5:5) for 24 h and then transferred to 70% ethanol for at least 24 h, then spikelets were dissected and pistils were cleared following the protocol described by Young et al. (1979) [92]. The ovaries were observed with a Leica DM2500 light transmission microscope equipped with a differential interference contrast (DIC) system and a digital camera (Leica Microsystems DFC 295. Wetzlar, Germany). At least 47 ovaries per plant (mean number of 73) were analyzed for the presence of single meiotic embryo sacs (MES), single or multiple AES, or one MES plus one or more AES (MES + AES), aborted ES (AbES) were also recorded. In individuals with a low number of ovaries containing AES (less than 0.05%); the number of ovaries examined was increased up to at least 134. Apospory expressivity was estimated as the percentage of ovaries containing at least one AES (alone or in combination with MES), out of the total number of ovaries scored. Embryo sac types were classified according to the method described by Norrmann et al. (1989) [55]. In particular, embryo sacs showing the egg apparatus, two polar nuclei, and a cluster of antipodal cells were classified as meiotic of the *Polygonum* type. Embryo sacs showing the egg apparatus and two polar nuclei, but lacking antipodal cells, were considered AESs of the *Paspalum* type [67]. The environmental effects of the different locations were evaluated by quantifying the %AES in anthesis ovaries fixed in Corrientes and Zavalla. The temporal stability of the trait was estimated by fixing the ovaries in different years in Zavalla.

### 4.3. AFLP Markers

DNA was extracted by using the CTAB method according to Martínez et al. (2003) [19]. Library construction was performed following the protocol described by Vos et al. (1995) [93] with modifications reported in Cnops et al. (1996) [94]. Briefly, genomic DNA (300 ng) was digested and ligated at 37 °C for 4 h using 5 U *Eco*-RI, 5 U *Mse*-I (New England Biolabs, Inc., Ipswich, MA, USA), 25 pmol *Mse*-I adapter, 2.5 pmol *Eco*-RI adapter, 0.2 mM ATP, 1 U T4 ligase (New England Biolabs Inc., Ipswich, MA, USA), and 1X buffer NEB CutSmart^®^ buffer (New England Biolabs Inc., Ipswich, MA, USA). Two consecutive amplification reactions were performed. A dilution (1:10) of digested and ligated DNA (5 µL) was pre-amplified mixing with 75 ng of each of three different primer combinations (Eco+C/Mse+C, Eco+A/Mse+C or Eco+C/Mse+A), 1.5 mM MgCl_2_, 10 mM Tris-HCl, 0.2 mM dNTPs (Pharmacia Biotech, Staffanstorp, Sweden), 1 U Taq DNA polymerase (Thermo Scientific, Waltham, MA, USA) and 1X buffer (Thermo Scientific, Waltham, MA, USA). Cycling conditions to ensure optimal primer amplification were: 72 °C for 5′, 1 cycle of 45″ at 94 °C, 30″ at 65 °C, 1′ at 72 °C followed by 12 cycles decreasing annealing temperature by 0.7 °C each cycle and 22 cycles of 30″ at 94 °C, 30″ at 55.9 °C, 1 min at 72 °C and a final step of 5 min at 72 °C. Selective amplifications were performed by mixing dilutions (1:10) of pre-amplified DNAs (5 µL), with 33 different combinations of selective primers (Supplementary Materials Table S2), 20 ng of Eco + 3 primer (labeled with 6-FAM, Sigma-Aldrich), 20ng Mse + 3 primer (Sigma-Aldrich, Burlington, MA, USA), 1.5 mM MgCl_2_, 0.2 mM dNTPs (Sigma-Aldrich, Burlington, MA, USA), 0.4 U Taq DNA polymerase (Thermo Scientific, Waltham, MA, USA), and 1X buffer (Thermo Scientific, Waltham, MA, USA). Cycling conditions to ensure optimal primer selectivity were: four cycles of 45″ at 94 °C. 30′ at 65 °C, 1′ at 72 °C followed by 12 cycles decreasing annealing temperature by 0.7 °C each cycle and 22 cycles as mentioned before. Each product (1 µL) was mixed with 10 µL of formamide and 0.1 µL of standard size (Genescan LIZ 500, Thermo Scientific, Waltham, MA, USA), then were denatured at 94 °C for 5′. Samples were analyzed with the ABI 3130xl Genetic Analyzer (Thermo Scientific, Waltham, MA, USA) using Genemapper 4.0 software. The F_1_ hybrid condition was verified by AFLP during the development of the linkage map. Eighty-seven individuals, out of a total of 88, from which good quality AFLP libraries were obtained, were found to have pollen donor-specific bands. Each plant had at least 98 bands of the paternal genotypes, and no plants of apomictic or self-fertilized origin, with only maternal bands, were detected (Supplementary Materials Table S7).

### 4.4. Genetic Linkage Analysis

Segregation data from each parental genotype were analyzed independently. As the diploid F_1_ mapping populations come from non-inbred parents, different allelic configurations were expected for each locus [95]. For single dose amplification fragments (SDAFs), which are polymorphic between parents (i.e., present in R6#45 and absent R5#49 and vice versa), the expected segregation ratio was 1:1. Markers that were monomorphic between parents, but segregating in the population, were termed Bi-parental Single Dose Fragments (BSDF) and their expected segregation ratio was 3:1 for presence:absence respectively. A χ2 test was used to determine the goodness of fit between the observed and the expected number of genotypes for each segregation class. For linkage analysis, each parental data file included both SDAF and BSDF markers. Two genetic linkage maps were constructed, one for each parental genotype, using JoinMap 4.1 software [96] and selecting the cross-pollinator full-sib population (CP) option. Bi-parental markers were used as allelic bridges to identify homologous linkage groups (LG) between the parental genotypes [95]. Markers with identical segregation in different individuals (>98%) were removed using the JoinMap 4.0 command “similarity of individuals”. Clustering analysis was carried out using a LOD score threshold of 6.0 or higher and a default linkage value of 0.4 for recombination frequency, the maximum detectable recombination fraction (maxR). Marker order and genetic distance within each linkage group were calculated using a regression mapping algorithm and the mapping function of Kosambi [97] with default options. Subsequently, a second set of markers was added to the originally established groups with a LOD score < 2.0 using the “fixed order” function. In addition, the segregation data for the presence/absence of those markers linked in the “repulsion phase” were re-coded (inverted) to include them in one linkage group per homologous pair according to al-Janabi et al. (1993) [98].

### 4.5. Linear Regression Analysis

To identify the markers associated with the trait, a linear regression analysis was performed using molecular markers as an independent variable and the %AES as a dependent variable [71]. Each F_1_ sub-population, 37 individuals from the F_1-C_ and 47 individuals from F_1-Z_, was analyzed separately due to the variation of the trait, verified in the present work, between the two locations. As apospory expressivity did not have a normal distribution (Shapiro–Wilk’s test showed a < 0.05), the data were transformed (Y = Log (%AES + 1)) reaching normal distribution (*p* = 0.95 and 0.34 for Corrientes and Zavalla, respectively). Markers were considered to be associated when *p* < 0.05, the R^2^ was understood as the proportion of phenotypic variation explained by each QTL [71].

### 4.6. Statistical Analysis

Quantitative comparison between %AES of different genotypes or between the same genotype grown under different conditions was performed according to Delgado et al. (2016) [69]. In brief, confidence intervals (CIs), around the AES frequency of each plant, were calculated using the online resource http://vassarstats.net/ (accessed on 17 August 2021). [99], following the method described by Wilson [100], without continuity correction. Fisher´s exact test [101] was used to determine the differences between the AES frequency of the experimental samples; calculations were performed using the online resource MeasuringU (https://measuringu.com/calculators/ab-cal/) (accessed on 17 August 2021). [102] to compare independent proportions, while the non-parametric Mood median test, using STATGRAPHIC Centurion XVI (Version 16.1.03), was used to compare the median of the frequency of apospory between F_1_ and F_2_ progenies.

## 5. Conclusions

Here, we report that apospory expressivity is influenced by the environment in both diploid and tetraploid genotypes of *P. rufum*. This is an important aspect to be considered for future application of apomixis technology and its introduction into sexual species. Our results show that it is possible to transfer the degree of apospory expressivity from parents to the offspring. The marker analysis allowed us to generate the first linkage map for the diploid system of *P. rufum* and to identify at least one 5.7 cM genomic region, in an LG, significantly associated with apospory expressivity. The molecular tools developed will be essential in the search for markers associated with other apomictic components from a qualitative and quantitative perspective in the diploid system of *P. rufum*.

## Figures and Tables

**Table 1:** Description of the genotypes used in the present work that had been previously characterized in Corrientes.

Individual	Ploidy Level	%AES	Nº of Scored Ovules ^1^	Origin	Reference
R6#45	2*x*	5.77	104	Paso Lucero, Corrientes, Argentina	Sartor et al., (2011), Delgado et al. (2014)
R5#49	2*x*	13.3	123	Saladas, Corrientes, Argentina	Sartor et al., (2011), Delgado et al., (2014)
F_1-C_#9	2*x*	1.22	82	R6#45 × R5#49	Delgado et al. 2016
F_1-C_#12	2*x*	0.00	112	R6#45 × R5#49	Delgado et al. 2016
F_1-C_#31	2*x*	22.22	36	R6#45 × R5#49	Delgado et al. 2016
F_1-C_#15	2*x*	32.76	58	R6#45 × R5#49	Delgado et al. 2016
F_1-C_#39	2*x*	35.85	53	R6#45 × R5#49	Delgado et al. 2016
LD1	4*x*	31.94	72	R6#49 ^2^	Delgado et al. 2016
LD3	4*x*	24.59	61	R6#45 × R2#18 ^3^	Delgado et al. 2016
Q3756	4*x*	96.1	103	Uruguay: unknown locality	Norrmann et al. 1989

^1^ Infloresences fixed in Corrientes; ^2^ colchicine treatment of cariopses obtained by open pollination; ^3^ colchicine treatment of cariopses obtained by crossing R6#45 × R2#18.

**Table 2:** Cytoembryological analysis of *P. rufum* diploid hybrids from F_1-Z_ population characterized in Zavalla.

Individuals	Ovary Number						
	Total	AbES	SES	AES	SES + AES	NC	%AES ^1^
F_1-Z_#67	118	33	84	0	0	1	0.00
F_1-Z_#74	132	81	43	0	0	8	0.00
F_1-Z_#89	104	2	102	0	0	0	0.00
F_1-Z_#91	117	17	97	0	1	2	0.85
F_1-Z_#93	109	24	84	0	1	0	0.92
F_1-Z_#55	105	4	100	0	1	0	0.95
F_1-Z_#79	103	3	98	0	1	1	0.97
F_1-Z_#83	99	1	97	0	1	0	1.01
F_1-Z_#71	90	2	87	0	1	0	1.11
F_1-Z_#45	73	13	57	0	1	2	1.37
F_1-Z_#52	142	76	59	0	2	5	1.41
F_1-Z_#73	115	6	107	0	2	0	1.74
F_1-Z_#69	105	53	50	0	2	0	1.90
F_1-Z_#63	97	2	92	0	2	1	2.06
F_1-Z_#66	92	30	49	0	2	11	2.17
F_1-Z_#47	106	30	71	0	3	2	2.83
F_1-Z_#90	103	6	64	0	3	30	2.91
F_1-Z_#87	100	4	91	0	3	2	3.00
F_1-Z_#53	99	5	91	0	3	0	3.03
F_1-Z_#68	98	5	89	0	3	1	3.06
F_1-Z_#85	98	2	93	0	3	0	3.06
F_1-Z_#81	130	21	102	0	4	3	3.08
F_1-Z_#61	96	2	90	0	3	1	3.13
F_1-Z_#58	94	6	83	0	3	2	3.19
F_1-Z_#48	92	6	83	0	3	0	3.26
F_1-Z_#84	89	3	83	0	3	0	3.37
F_1-Z_#57	100	5	78	0	4	13	4.00
F_1-Z_#44	98	52	35	2	2	7	4.08
F_1-Z_#59	95	1	55	0	4	35	4.21
F_1-Z_#77	117	44	68	0	5	0	4.27
F_1-Z_#62	100	8	83	0	5	4	5.00
F_1-Z_#49	76	4	67	0	4	1	5.26
F_1-Z_#50	112	3	103	0	6	0	5.36
F_1-Z_#70	110	16	88	0	6	0	5.45
F_1-Z_#51	72	3	65	0	4	0	5.56
F_1-Z_#56	114	4	103	0	7	0	6.14
F_1-Z_#75	108	6	93	0	7	2	6.48
F_1-Z_#82	108	10	90	0	7	1	6.48
F_1-Z_#76	86	28	52	0	6	0	6.98
F_1-Z_#78	78	0	72	0	6	0	7.69
F_1-Z_#64	103	6	88	0	8	1	7.77
F_1-Z_#92	112	1	101	0	9	1	8.04
F_1-Z_#88	94	0	86	0	8	0	8.51
F_1-Z_#65	105	3	93	0	9	0	8.57
F_1-Z_#54	109	4	95	0	10	0	9.17
F_1-Z_#60	82	9	64	0	9	0	10.98
F_1-Z_#46	47	3	37	0	6	1	12.77
F_1-Z_#72	79	1	67	1	10	0	13.92
F_1-Z_#86	95	0	78	0	17	0	17.89
R6#45 ^2^	222	3	211	0	8	0	3.60
R5#49 ^2^	265	67	172	3	14	9	6.42
F_1-Z_ Mean (S.D.)							4.56 (3.82) *
F_1-C_ Mean (S.D.)							9.31 (8.03) ^3^

AbES: aborted embryo sacs; SES: sexual embryo sacs; AES: aposporous embryo sacs; NC no classified; ^1^ percentages of ovaries carrying AES over the total ovaries analyzed; ^2^ parental genotypes measured in 2016; S.D.: Standard deviation; ^3^ values taken from Delgado et al., 2016; * statistically significant differences between mean values of Zavalla and Corrientes.

**Table 3:** Apospory expressivity of parental genotypes and F_2H_ (#15 × #39) progeny.

	Individual		% AES (n)		% AES (n)	Mean (S.D)
Parental genotypes	F_1-C_#15	2017	6.97 (215)	2018	9.90 (101)	8.79 (1.81) ^1^
	F_1-C_#39		9.20 (239)		9.33 (75)	
F_2L_ #35 × #39	F_2H_#7	2016	2.7(73)	2017	0.9 (111) ^ns^	1.82 (0.01)
	F_2H_#5		6.3 (112)		7.7 (78) ^ns^	6.97 (0.01)
	F_2H_#6		6.8 (88)		5.0 (100) ^ns^	5.91 (0.01)
	F_2H_#4		7.6 (118)		6.5 (107) ^ns^	7.08 (0.01)
	F_2H_#3		10.8 (102)		9.4 (96) ^ns^	10.08 (0.01)
	F_2H_#1		12.4 (105)		9.9 (81) ^ns^	11.13 (0.02)
	F_2H_#9		13.5 (74)		6.0 (100) ^ns^	9.76 (0.05)
	F_2H_#8		13.6 (103)		4.8 (124) *	7.27 (0.03)
	F_2H_#2		16.7 (96)		14.5 (55) ^ns^	15.61 (0.01)
F_2L_ Mean (S.D.)			10.05 (4.47)		7.19 (3.83)	8.62 (4.29)

n: Number of ovaries analyzed;e S.D.: Standard deviation; ^ns^ non-significant difference between both years, at *p* < 0.05;e * seignificant difference, *p* < 0.05; ^1^ iencluding %AES of F_1-C_#15 and F_1-C_#39 measured in Zavalla in 2017 and 2018.

**Table 4:** Apospory expressivity of parental genotypes and F_2L_ (#9 × #12) progeny.

	Individual		% AES (n)		% AES (n)	Mean (S.D)
Parental genotypes	F_1-C_#12	2017	0.00 (168)	2018	0.00 (128)	1.40 (1.98) ^1^
	F_1-C_#9		N.D.		2.76 (145)	
F_2L_ #9 × #12	F_2L_#1	2016	0 (95)	2017	0 (111) ^ns^	0 (0)
	F_2L_#6		1.1 (94)		0 (81) ^ns^	0.53 (0.75)
	F_2L_#9		2.2 (90)		2.1 (94) ^ns^	2.17 (0.07)
	F_2L_#2		0.9 (110)		1.1 (92) ^ns^	1 (0.13)
	F_2L_#8		1 (101)		1.1 (92) ^ns^	1.04 (0.07)
	F_2L_#5		0.9 (109)		2.5 (79) ^ns^	1.72 (1.14)
	F_2L_#7		0 (60)		2.9 (102) ^ns^	1.47 (2.08)
	F_2L_#3		7 (71)		5.4 (111) ^ns^	6.22 (1.16)
	F_2L_#10		16.9 (83)		9.7 (113) ^ns^	13.3 (5.04)
F_2L_ Mean (S.D.)			3.33 (5.51)		2.75 (3.09)	3.05 (4.34)

n: Number of ovaries analyzed; S.D.: Standard deviation; ^ns^ non-significant difference between both years, at *p* < 0.05. ^1^ Including only %AES of F_1-C_#12 and F_1-C_#9 measured in Zavalla in 2018.

**Table 5:** Details of AFLP markers segregation in mapping population.

Parental	Number of Primer Combination	Number of Markers	Marker Segregation		
			SDAF (1:1)	BSDF (3:1)	Distorted ^1^
R6#45	33	278	266	-	12
R5#49	33	239	221	-	18
R6#45-R6#49	33	110	-	94	16

^1^ Segregation was considered distorted at *p* < 0.05.

**Table 6:** Markers significantly associated with apospory expressivity detected in the F_1-C_ population.

Marker	Type of Marker	LG (M/P)	Position (cM) (M/P)	b Parameter ^1^	R^2^	*p*-Value
C25_263.9	Maternal	2-M	14.5	0.29	0.21	0.004
B4_490.5	Paternal	3-P	104.7	0.24	0.13	0.027
C5_411.5	Maternal	3-M	16.5	−0.22	0.12	0.032
C35_334	Maternal	3-M	48.9	−0.23	0.13	0.025
B5_360.1	Maternal	3-M	52.2	−0.25	0.15	0.018
C4_227.4	Maternal	3-M	54.4	−0.22	0.11	0.043
C32_150.4	Maternal	3-M	56.7	−0.23	0.13	0.025
C33_187.4	Maternal	3-M	61.4	−0.23	0.13	0.026
XC31_2201	Biparental	4-M/4-P	0.0/36.67	0.42	0.20	0.005
XB4_324.5	Biparental	4-M/4-P	18.8/56.65	0.27	0.12	0.036
C23_279.7	Paternal	4-P	75.2	0.21	0.10	0.049
B1_236.6	Maternal	4-M	21.6	−0.22	0.34	0.042
C32_244.8	Maternal	4-M	37.7	0.29	0.19	0.006
C25_292.5	Maternal	4-M	44.4	−0.21	0.10	0.047
C33_384.1 (B16_164.6) ^2^	Maternal	4-M	48.1	−0.22	0.12	0.036
C17_402.3	Maternal	4-M	66.6	0.24	0.13	0.025
B1_72.8	Maternal	5-M	47.2	−0.23	0.13	0.030
C5_221.1	Paternal	10-P	0.0	0.22	0.12	0.035
B3_217.8	Paternal	10-P	4.6	0.22	0.12	0.035
C29_141.3	Paternal	10-P	10.8	0.22	0.11	0.038
C43_342.72 (C29_332.2) ^2^	Maternal	4-M	38.3	−0.29	0.19	0.006
C8_142.52 (C32_244.8) ^2^	Maternal	4-M	37.7	0.25	0.15	0.016
C29_151.42	Paternal	10-P	10.8	0.22	0.11	0.038
B48_256.83	Paternal	Unmapped	-	0.22	0.12	0.032
C5_194.8	Paternal	Unmapped	-	0.22	0.12	0.035
XC5_1093	Biparental	Unmapped	-	0.25	0.11	0.039

M/P: maternal/paternal; ^1^ slope of the linear regression function marker eliminated by similarity; ^2^ the specifications shown correspond to the similar mapped markers (between brackets). Markers with R^2^ ≥ 0.15 are highlighted in grey.

**Table 7:** Markers significantly associated with apospory expressivity detected in the F_1-Z_ population.

Marker	Type of Marker	LG (M/P)	Position (cM) (M/P)	b Parameter ^1^	R^2^	*p*-Value
XC4_1399	Biparental	1-P	52.1	0.24	0.12	0.02
C4_173.5	Maternal	3-M	25.9	0.22	0.12	0.02
C17_260.6	Paternal	3-P	30.7	0.20	0.10	0.02
C17_261.7	Paternal	3-P	27.0	0.21	0.11	0.02
B5_193.1	Maternal	3-M	101.2	0.24	0.15	0.01
XC4_2241	Biparental	3-M/3-P	111.89/37.5	0.27	0.16	0.00
XC25_3915	Biparental	6-P	2.9	−0.27	0.11	0.02
B54_74.7	Paternal	6-P	66.0	−0.19	0.10	0.03
C4_120.6	Maternal	7-M	55.6	0.22	0.12	0.02
B54_309.9	Maternal	7-M	71.3	−0.26	0.17	0.00
C23_372.6	Maternal	8-M	71.8	−0.21	0.11	0.02
C31_336.52 (C12_83) ^2^	Maternal	10-M	28.7	0.25	0.14	0.01
C12_214.8	Maternal	Unmapped	-	0.23	0.13	0.01

M/P: maternal/paternal; ^1^ slope of the linear regression function; ^2^ marker eliminated by similarity, the specifications shown correspond to the similar mapped markers (between brackets). Markers with R^2^ ≥ 0.15 are highlighted in grey.

## Data Availability

All the data presented in this study are available in the article and in Supplementary Materials.

## Supplementary Materials

The following are available online at https://www.mdpi.com/article/10.3390/plants10102100/s1. Figure S1: Amplification profiles of RAPD markers; Figure S2: Amplification profiles of AFLP markers obtained using capillary electrophoresis with the ABI 3130xl sequencer; Table S1: Cytoembryological analysis of *P. rufum* diploid hybrids and tetraploid genotypes in Zavalla; Table S2: AFLP primers sequences and combinations used to build the genetic linkage maps; Table S3: Specifications of maternal and paternal map; Table S4: List of bi-parental markers shared between R6#45 and R5#49 maps; Table S5: UBC primer used to verify hybridity in F_2_ progenies; Table S6: Segregations of RAPD markers in F_2_ offsprings and Table S7: Single dose AFLP markers detected in each F_1_ offspring.
