# Supplementary material for: Environmental and Genetic Factors Affecting Apospory Expressivity in Diploid Paspalum rufum
Source: Plants (Basel). 2021 Oct 4;10(10):2100. doi: 10.3390/plants10102100 (PMC8537111; doi:10.3390/plants10102100)
Supplement: Supplementary file 1 [file plants-10-02100-s001.zip › plants-1377678-supplementary.pdf]

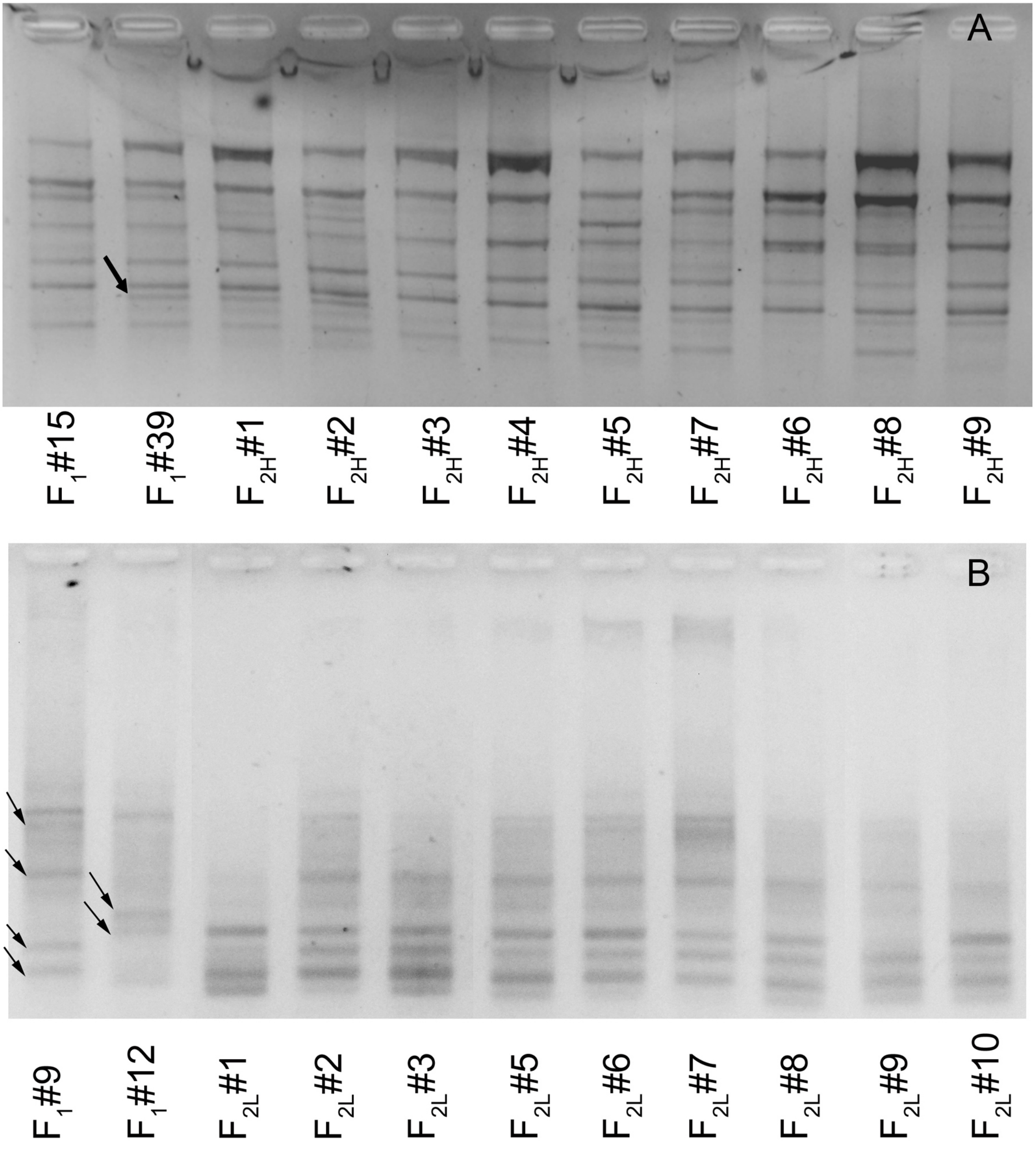

Figure S1. Amplification profiles of RAPD markers. A) Example of ubc349 primer amplification profile in the F_{2H} population. B) Example of ubc301 primer amplification profile in the F_{2L} population. Black arrows indicate polymorphic bands.

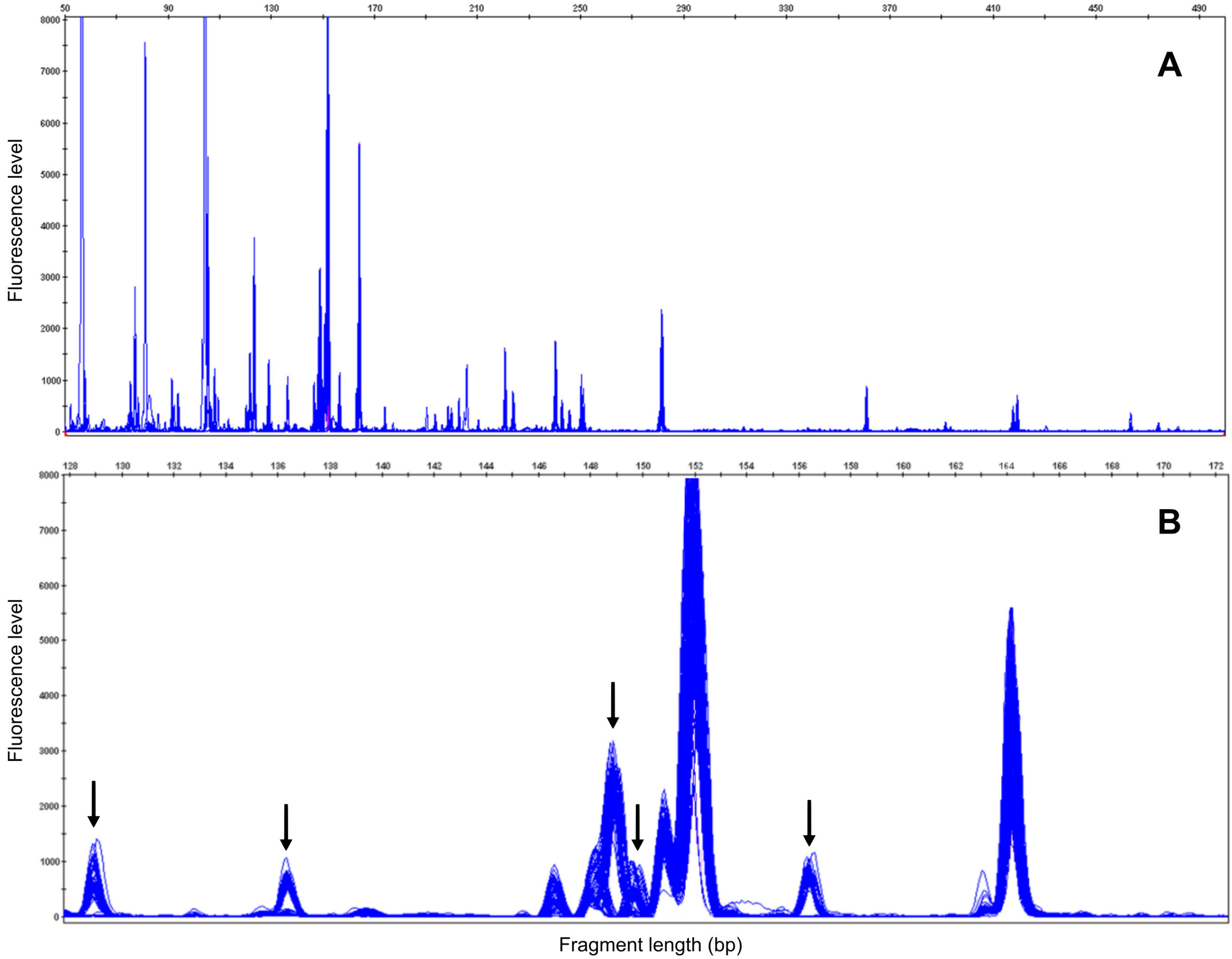

Figure S2. Amplification profiles of AFLP markers obtained using capillary electrophoresis with the ABI 3130xl sequencer, visualised with the Genetic Analyzer software (Life technologies). A) Detail of the amplification profile, over the full F1 population, of the EcoRICAC-MseICTG primer combination in the range of 50-500 bp. B) Detail of an amplification profile viewed on an enlarged scale. Black arrows indicate peaks representing a polymorphic marker.

Table S1. AFLP primer sequences and combinations used to build the genetic linkage maps

	Primer sequences (5' - 3')	
Combination	Eco + 3nt primer sequence	Mse + 3nt primer sequence
C1	GACTGCGTACCAATTCAGC	GATGAGTCCTGAGTAACCAA
C4	GACTGCGTACCAATTCAGA	GATGAGTCCTGAGTAACCAA
C5	GACTGCGTACCAATTCAGA	GATGAGTCCTGAGTAACAGA
C7	GACTGCGTACCAATTCCAG	GATGAGTCCTGAGTAACCAG
C8	GACTGCGTACCAATTCCAG	GATGAGTCCTGAGTAACCAG
C12	GACTGCGTACCAATTCCAC	GATGAGTCCTGAGTAACCAA
C13	GACTGCGTACCAATTCACT	GATGAGTCCTGAGTAACCTG
C17	GACTGCGTACCAATTCACA	GATGAGTCCTGAGTAACCAA
C20	GACTGCGTACCAATTCCCA	GATGAGTCCTGAGTAACCAA
C22	GACTGCGTACCAATTCCAA	GATGAGTCCTGAGTAACCTG
C23	GACTGCGTACCAATTCCAA	GATGAGTCCTGAGTAACCAA
C24	GACTGCGTACCAATTCCAA	GATGAGTCCTGAGTAACCAC
C25	GACTGCGTACCAATTCCAC	GATGAGTCCTGAGTAACAAC
C29	GACTGCGTACCAATTCCAC	GATGAGTCCTGAGTAACAGA
C31	GACTGCGTACCAATTCCAC	GATGAGTCCTGAGTAACATC
C32	GACTGCGTACCAATTCCCA	GATGAGTCCTGAGTAACAAC
C33	GACTGCGTACCAATTCCCA	GATGAGTCCTGAGTAACACA
C35	GACTGCGTACCAATTCCCA	GATGAGTCCTGAGTAACACG
C42	GACTGCGTACCAATTCCAA	GATGAGTCCTGAGTAACACG
C43	GACTGCGTACCAATTCCAA	GATGAGTCCTGAGTAACAGA
B1	GACTGCGTACCAATTCAGC	GATGAGTCCTGAGTAACCGA
B2	GACTGCGTACCAATTCAGC	GATGAGTCCTGAGTAACCCC
B3	GACTGCGTACCAATTCAGC	GATGAGTCCTGAGTAACCGC
B4	GACTGCGTACCAATTCAGC	GATGAGTCCTGAGTAACCTT
B5	GACTGCGTACCAATTCAGA	GATGAGTCCTGAGTAACCGA
B7	GACTGCGTACCAATTCAGA	GATGAGTCCTGAGTAACCGC
B13	GACTGCGTACCAATTCACA	GATGAGTCCTGAGTAACCGA
B16	GACTGCGTACCAATTCACA	GATGAGTCCTGAGTAACCTT
B23	GACTGCGTACCAATTCACC	GATGAGTCCTGAGTAACCTT
B32	GACTGCGTACCAATTCCCA	GATGAGTCCTGAGTAACCGA
B48	GACTGCGTACCAATTCCAG	GATGAGTCCTGAGTAACACA
B53	GACTGCGTACCAATTCCAG	GATGAGTCCTGAGTAACATC
B54	GACTGCGTACCAATTCCGG	GATGAGTCCTGAGTAACAAC

Table S2: Single dose AFLP markers detected in each F_1 offspring

F_1 genotype	Number of SDAF AFLP markers		
	Maternal	Paternal	total
1	145	114	259
2	150	124	274
3	127	107	234
4	147	104	251
5	139	120	259
6	122	117	239
7	146	122	268
8	129	98	227
9	145	110	255
10	126	105	231
12	143	122	265
13	150	122	272
15	132	128	260
16	144	130	274
17	151	117	268
18	125	118	243
19	143	119	262
20	130	119	249
21	144	132	276
22	137	130	267
23	133	147	280
24	133	119	252
25	124	106	230
26	127	137	264
27	148	113	261
29	145	126	271
30	142	122	264
31	115	128	243
33	136	120	256
34	158	131	289
35	139	136	275
36	139	121	260
37	129	119	248
38	143	117	260
39	136	127	263
40	151	115	266
41	155	126	281
42	129	122	251
43	142	120	262
44	139	122	261
45	150	109	259
46	150	108	258
47	141	112	253
48	144	116	260
49	134	105	239
50	150	110	260
51	115	129	244
52	149	114	263
53	138	114	252
54	128	112	240
55	148	112	260
56	143	119	262
57	138	102	240
58	139	134	273
59	151	117	268
60	135	121	256
61	148	143	291
62	144	119	263
63	137	124	261
64	143	107	250
65	142	123	265
66	129	123	252
67	137	108	245
68	134	120	254
69	135	116	251
70	147	109	256
71	141	122	263
72	148	102	250
73	143	120	263
74	127	123	250
75	125	123	248

76	120	116	236
77	123	118	241
78	154	116	270
79	144	110	254
81	129	113	242
82	137	121	258
83	135	106	241
84	128	119	247
85	138	99	237
86	123	115	238
87	133	115	248
88	147	113	260
89	139	122	261
91	143	109	252
92	143	128	271
93	138	121	259

Table S3: UBC primer used to verify hybridity in F_2 offsprings

Number	Sequence
301	CGG TGG CGA A
310	GAG CCA GAA G
329	GCG AAC CTC C
344	TGT TAG GCA C
349	GGA GCC CCC T

Table S4: Segregations of RAPD markers in F_2 offsprings

		Parental		F_2 offspring									
	Marker	F_1#9	F_1#12	F_{2L}#1	F_{2L} #2	F_{2L} #3	F_{2L} #5	F_{2L} #6	F_{2L} #7	F_{2L} #8	F_{2L} #9	F_{2L} #10
F_{2L} 9x12	ubc301 (a)[1]	-	+	+	+	+	+	+	+	+	-	+
	ubc301 (b)[1]	-	+	+	+	+	+	+	-	+	+	+
	ubc310	-	+	-	+	+	N.D.	+	+	-	+	+
	ubc349	-	+	+	+	+	-	+	+	N.D.	+	+
		F_1#15	F_1#39	F_{2H} #1	F_{2H} #2	F_{2H} #3	F_{2H} #4	F_{2H} #5	F_{2H} #6	F_{2H} #7	F_{2H} #8	F_{2H} #9
F_{2H} 15x39	ubc310 (a)[1]	-	+	+	+	+	+	+	-	+	+	+
	ubc310 (b)[1]	-	+	+	+	-	-	+	+	+	+	N.D.
	ubc349	-	+	+	+	-	-	+	-	+	+	+
	ubc329	-	+	+	+	-	-	-	-	-	+	-
	ubc344	-	+	+	+	+	+	+	+	+	+	+

[1]Indicate different polymorphic bands from the same RAPD marker
N.D. no detected band.

Table S5: Cytoembryological analysis of *P. rufum* diploid hybrids and tetraploid genotypes in Zavalla

Individuals	Ploidy level	Campaing	Ovary number						%AES[1]
			Total	AbES	SES	AES	SES+AES	NC	
F_{1-Z}#89	2x	2017	104	2	71	0	0	0	0.00
		2018	89	18	64	0	0	7	0.00
F_{1-Z}#67	2x	2017	118	33	57	0	0	0	0.00
		2018	83	5	78	0	0	0	0.00
F_{1-Z}#52	2x	2017	142	76	59	0	2	5	1.41
		2018	95	19	35	0	0	0	0.00
F_{1-C}#31	2x	2018	104	0	93	0	10	1	9.62
LD1	4x	2018	53	0	47	0	6	0	11.32
LD3	4x	2018	47	0	40	0	7	0	14.89
3756	4x	2018	72	0	24	33	15	2	66.67

AbES: aborted embryo sacs; SES: sexual embryo sacs; AES: aposporous embryo sacs; NC: no classified.
[1] Percentages of ovaries carrying AES over the total ovaries analyzed. Grey files highlight genotypes that were statistically compared between each other.

Table S6: Specifications of maternal and paternal map

Specification types		Maternal map	Paternal map
Total number of LGs		10	10
N° of markers	Total	360	314
	Highly similar	75	74
	Mapped	234	212
	Mean per LG	23.4 (± 11.7)	21.2 (± 11.8)
	Maximum in a LG	44	50
	Minimum in a LG	6	10
	In the largest LG	33	27
	In the shortest LG	10	14
Distance (cM)	Total coverage	1071.8	914
	Largest LG coverage	151.9	117.2
	Shortest LG coverage	62.7	46.7
	Mean between markers	4.78 (±4.8)	4.53 (± 5.4)
	Maximum between markers	28.74	34.9

Table S7: List of biparental markers shared between R6#45 and R5#49 maps

GL.	1	2	3	4	5	7	8	9
	XB48_384	XB16_159,2	XC32_1489	XB1_165	XC4_1798	XC12_1869	XB7_122,5	XC43_2561
	XC43_587	XC42_1869	XC4_2241	XB4_324,5	XC7_1639	XC4_4884	XC1_4177	
	XC12_4916	XC13_2463	XC1_1203	XC31_2201	XC4_179	XC17_2317	XC12_4247	
	XC32_737			XB4_102,8	XC13_1565	XC33_716	XC35_4467	
	XC31_4072			XB13_125,2	XB13_284,2	XC23_997	XC23_2433	
	XC12_2438				XC31_1354			
	XC12_2422				XC33_1346			
	XC24_825				XB13_111			
					XB5_132,3			
					XC1_129			
					XC24_2577			
					XC42_4392			
